# Ischemic fallopian tube necrosis with hydatid of Morgagni secondary to post‑caesarean intra‑abdominal adhesions entrapment in the 36th week of pregnancy: A case report and mini‑review of the literature

**DOI:** 10.3892/mi.2025.231

**Published:** 2025-04-01

**Authors:** Anna Thanasa, Efthymia Thanasa, Ioannis-Rafail Antoniou, Emmanouil Xydias, Apostolos Ziogas, Alexandros Leroutsos, Athanasios Chasiotis, Ioannis Paraoulakis, Ioannis Thanasas

**Affiliations:** 1 Department of Health Sciences, Medical School, Aristotle University of Thessaloniki, 54124 Thessaloniki, Greece; 2 Department of Obstetrics and Gynecology, General Hospital of Trikala, 42100 Trikala, Greece; 3 Department of Obstetrics and Gynaecology, EmbryoClinic IVF, 55133 Thessaloniki, Greece; 4 Department of Medicine, University of Thessaly, School of Health Sciences, 41334 Larissa, Greece

**Keywords:** cesarean section, intra-abdominal surgical adhesions, tubal entrapment, ischemia, necrosis, preterm delivery, diagnosis, management

## Abstract

Surgical adhesions in the pelvis are a well-known complication following cesarean sections. The present study describes a unique case involving acute intra-abdominal inflammation due to ischemic necrosis of the fallopian tube with a hydatid of Morgagni, resulting from entrapment in post-operative adhesions and leading to preterm labor. The patient described herein, a woman in her 36th week of pregnancy with a history of two prior lower-segment cesarean sections, presented with diffuse abdominal pain escalating in intensity and intermittent vomiting. A diagnosis of preterm labor was established, prompting a decision to proceed with cesarean section. The procedure was uneventful, revealing numerous post-operative pelvic adhesions intraoperatively. Further examination revealed ischemic necrosis of the left fallopian tube with a small hydatid of Morgagni, attributed to entrapment due to an adhesion, with no involvement of the left ovary. Upon dissecting the adhesion, tubal perfusion was promptly restored. A decision was made to preserve the affected fallopian tube and perform surgical drainage of the hydatid of Morgagni. Both the mother and newborn were discharged in excellent condition from the clinic on the 4th post-operative day. The case descrbied herein underscores the rarity of preterm labor resulting from ischemic tubal necrosis with a hydatid of Morgagni, secondary to post-cesarean section adhesions. It emphasizes the importance of an intraoperative examination for tubal and ovarian conditions to detect surgical adhesions and underscores the value of preoperative diagnosis and the prevention of post-operative adhesions in pregnant women undergoing cesarean section.

## Introduction

Cesarean section, when medically indicated, is a crucial procedure for safeguarding the lives of both the mother and newborn. However, the global rise in cesarean delivery rates, reaching levels as high as 30-40% in a number of countries (1), has brought attention to associated pathological conditions, such as dysmenorrhea, dyspareunia, chronic pelvic pain and infertility (2). Post-operative pelvic adhesions, a common complication following cesarean sections, particularly increase in rate and severity with each subsequent surgery (3). It has been found that the likelihood of adhesion formation escalates from 5% after the initial procedure to a notable 68% following three or more cesarean sections (4). The present study describes the case of a patient in which adhesions resulting from two prior cesarean sections led to the entrapment and ischemic necrosis of the left fallopian tube, with the presence of a small hydatid of Morgagni, resulting in preterm labor. While intra-amniotic inflammation is recognized as a predominant cause of preterm labor, accounting for an estimated 4-16% of all births (5) and a significant contributor to neonatal morbidity and mortality worldwide (6), the case described herein presents a unique scenario. The onset of preterm labor due to ischemic tubal necrosis with a hydatid of Morgagni, subsequent to entrapment in post-cesarean section adhesions, is an unprecedented occurrence in the English literature.

In light of this case, the authors aim to stress the importance of an intraoperative inspection of the fallopian tubes and ovaries during cesarean sections in order to detect and manage postoperative adhesions promptly. Additionally, the pre-operative identification of pelvic adhesions following cesarean delivery is crucial, and the present study aims to advocate for proactive measures to prevent adhesion formation in pregnant women with a history of cesarean sections, thereby reducing the risks of maternal and perinatal morbidity.

## Case report

A 24-year-old pregnant woman, having undergone two previous lower-segment cesarean sections, presented to the Emergency Department of the General Hospital of Trikala, Trikala, Greece during the 36th week of gestation with complaints of diffuse abdominal pain. She reported that the pain had commenced ~4 h prior, characterized by intermittent exacerbation and escalating intensity. The pain was diffuse across the abdomen, initially originating from the left lumbar region, where it was most intense. The pain was accompanied by multiple episodes of vomiting. Pain intensity was not associated with defecation and was unaffected by body posture. The patient denied any history of preterm labor, and her pregnancy had been uneventful thus far. A prenatal ultrasound and blood sugar monitoring yielded normal findings. Cervical length measurements via ultrasound remained within normal limits throughout each trimester. The patient exhibited a normal body mass index (20.8 kg/m^2^), and the healing process of her previous cesarean section scars was unremarkable, without any evidence of subcutaneous tissue stiffness, keloids, or hypertrophic scars. Additionally, there were no abdominal stretch marks indicative of pregnancy.

Upon a vaginal examination, cervical dilation (2 cm) was observed. A cardiotocography revealed uterine contractions. An obstetric ultrasound indicated normal fetal growth (estimated ultrasound weight, 2,560 g) and a normal amount of amniotic fluid (amniotic fluid index, 8 cm), with no ultrasonographic signs of placental abruption. A urinary tract ultrasound ruled out the presence of calculi and did not reveal any signs of obstructive uropathy (such as hydronephrosis). Laboratory tests indicated mild anemia (hematocrit level, 31.6%; hemoglobin level, 10.8 g/dl), persisting since the onset of pregnancy. The analysis of inflammation-related markers yielded negative results: White blood cells, 5,600/µl; neutrophils, 65.6%; C-reactive protein, 0.5 mg/dl. Coagulation, biochemical parameters and general urinalysis did not yield any notable findings. Vaginal fluid microscopy and culture revealed no microbial growth, and the test for detecting the premature rupture of fetal membranes yielded negative results.

**Figure 1:**
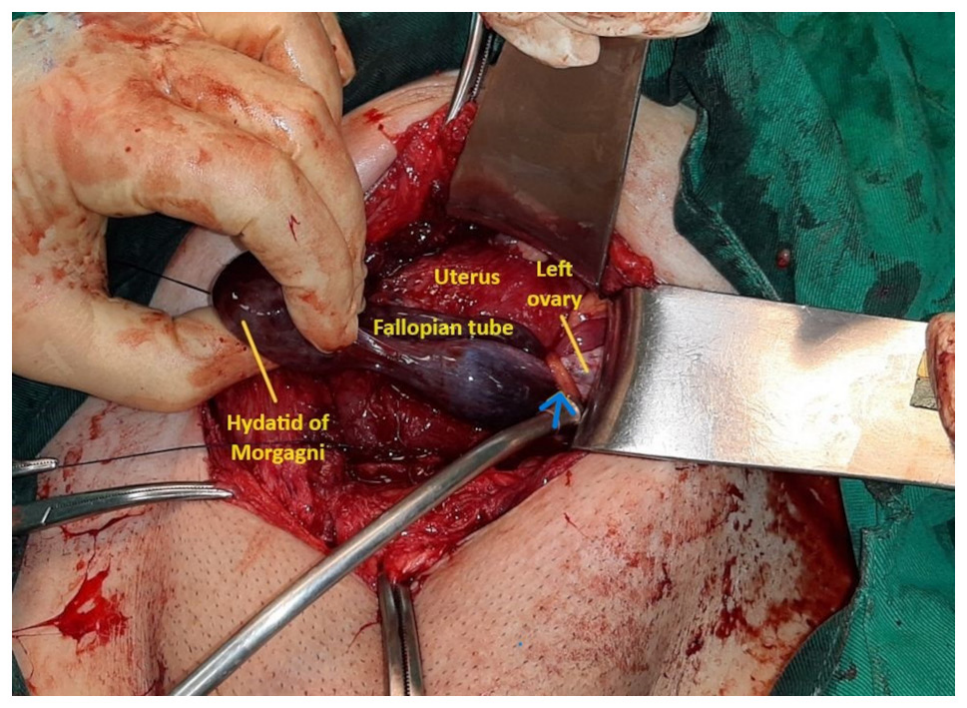
Intraoperative image illustrating the left fallopian tube and ovary upon inspection for intra-abdominal surgical adhesions following fetal delivery and closure of the uterine incision: Ischemic necrosis of the left fallopian tube and hydatid of Morgagni are evident, without the involvement of the left ovary. This condition is a consequence of their entrapment in a postoperative (post-cesarean section) adhesion (indicated by blue arrow).

**Figure 2:**
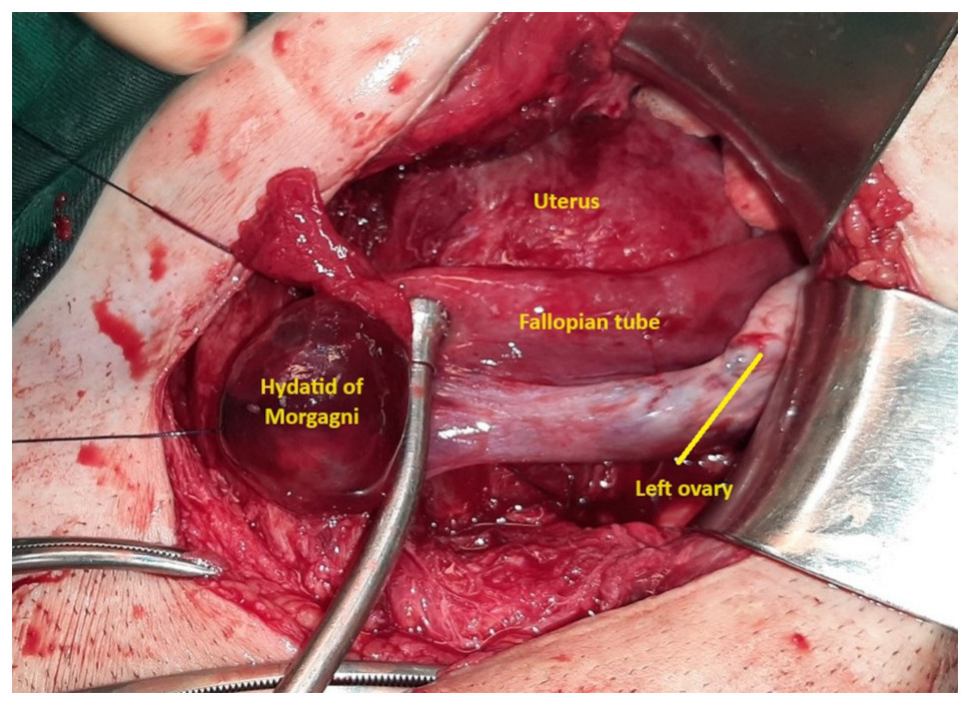
Intraoperative image of the left fallopian tube and ovary after dissection of the post-operative (post-cesarean section) adhesion: Immediate revascularization in the left adnexal area led to the decision to spare the inflamed and edematous affected fallopian tube and surgically drain the small hydatid of Morgagni.

Based on the clinical, ultrasound and laboratory findings outlined above, a diagnosis of preterm labor was established, necessitating the decision to proceed with a cesarean section. The General Hospital of Trikala lacked the logistical infrastructure (organized operating room near the delivery room with constant presence of an anesthesiologist) and expertise on the part of obstetrician-gynecologists required for attempting vaginal delivery following a previous cesarean section. Therefore, the patient, 2 days before completing 36 weeks of pregnancy, underwent a cesarean section, delivering a healthy, full-term female newborn weighing 2,490 g. The procedure was performed without complications, with normal blood loss. Intraoperatively, numerous post-operative adhesions were observed within the abdomen, particularly between the uterus and the bladder at the vesicouterine pouch, as well as between the uterus and the left lateral abdominal wall. During intraoperative abdominal examination, ischemic necrosis of the left fallopian tube with a small hydatid of Morgagni was noted, secondary to entrapment in post-cesarean section adhesions, with no involvement of the left ovary (Fig. 1). Upon the dissection of the adhesions, tubal perfusion was promptly restored within approximately two minutes (Fig. 2). It was decided to preserve the affected fallopian tube and perform surgical drainage of the small hydatid of Morgagni. The neonate did not require admission to the Neonatal Intensive Care Unit, and both the mother and newborn were discharged in excellent condition from the Obstetrics and Gynecology clinic of the General Hospital of Trikala on the 4th post-operative day. A follow-up gynecological assessment 10 weeks following delivery revealed normal findings from her physical and ultrasonographic examination.

## Discussion

The pre-operative diagnosis of intra-abdominal surgical adhesions resulting from previous cesarean sections poses a challenge. However, non-invasive methods, such as assessing the characteristics of the skin scar post-cesarean section, the presence of pregnancy stretch marks on the abdominal wall and utilizing ultrasound guidance to examine the uterine sliding point on the anterior abdominal wall have shown promise. The correct application and interpretation of these methods could significantly aid in pre-operative diagnosis before planned repeat cesarean sections (7). In the study by Tulandi *et al* (8) in 2011, it was demonstrated that pregnant women with keloids in the cesarean section scar were more likely to develop post-operative adhesions between the uterus and the bladder, as well as between the uterus and the abdominal wall. Recently, Seven *et al* (9) conducted a study in 2020, where they investigated the stiffness of subcutaneous tissue in previous cesarean section scars. Their findings revealed that elastographic assessment of subcutaneous tissue stiffness at the cesarean section scar site can predict the severity of intra-abdominal surgical adhesions preoperatively in repeat cesarean sections (9).

There is a notable association between abdominal wall stretch marks and intra-abdominal surgical adhesions in pregnant women with prior cesarean sections. In 2016, Dogan *et al* (10) conducted a study demonstrating that a higher score for abdominal wall stretch marks during pregnancy correlates with a reduction in intra-abdominal surgical adhesions among pregnant women scheduled for cesarean sections due to previous cesarean deliveries. Similarly, Cakir Gungor *et al* (11) concluded that the presence of pregnancy stretch marks on the abdominal wall could serve as a pre-operative indicator for predicting the formation of intra-abdominal surgical adhesions. In the case in the present study, despite the presence of multiple postoperative pelvic adhesions leading to entrapment and partial ischemic necrosis of the left fallopian tube, there was no association with the presence of keloids or subcutaneous tissue stiffness at the scar site, or pregnancy stretch marks on the abdominal wall.

In contrast to the aforementioned clinical indicators for the pre-operative prediction of intra-abdominal post-cesarean section adhesions, the absence of uterine sliding point on the anterior abdominal wall (limited uterine mobility) detected via ultrasound examination is considered the most significant and effective predictor of postoperative adhesions following previous cesarean sections (2). The recent study by Yosef *et al* (12) in 2023 concluded that ultrasound assessment of the uterine sliding point is a rapid, simple, and reliable method for prenatally predicting intraperitoneal adhesions. The aim is to adequately prepare pregnant women preoperatively and mitigate the risk of serious intraoperative and postoperative complications. The authors of that study reported a sensitivity of 100%, specificity of 86.84%, positive predictive value of 81.5%, negative predictive value of 100%, and accuracy of 91.67% for this method (12). Similar findings were reported by Charernjiratragul *et al* (13), who demonstrated that the uterine sliding point serves as an independent prognostic indicator of intra-abdominal surgical adhesions, with acceptable sensitivity, high specificity and negative predictive value. In the case described herein, the evaluation of the uterine sliding point on the anterior abdominal wall was positive, indicating no restriction of uterine mobility during an ultrasonographic examination. However, it is worth noting that the examination was performed by obstetricians-gynecologists at the Obstetrics and Gynecology Clinic of the General Hospital of Trikala, whose specialized skills in ultrasonography may be limited compared to specialized sonographers.

The prevention of intra-abdominal surgical adhesions in the pelvis may be facilitated by platelet-rich plasma infusion (14,15); however, further research on applied methodology is required to ascertain the value of such techniques in the context of prevention (16,17). Even surgical approach may aid in prevention, as the Misgav Ladach technique may be associated with a higher likelihood of post-cesarean section pelvic adhesion formation compared to more traditional methods, such as Pfannenstiel-Dörffler and low midline laparotomy-Dörffler techniques (18).

Performing a planned cesarean section in pregnant women with multiple post-operative pelvic adhesions is challenging and requires appropriate pre-operative preparation (19). In selected cases where the incision of the anterior uterine wall is not feasible due to severe pelvic adhesions, a low transverse incision in the posterior uterine wall after rotation may be a safe and effective approach (20). In the case in the present study, pelvic adhesions resulting from previous cesarean sections were not very severe. Entry into the peritoneal cavity and delivery of the fetus through an incision in the anterior uterine wall were achieved without significant difficulty. The challenges primarily stemmed from the lack of prenatal etiological diagnosis of preterm labor, which was established intraoperatively upon discovering ischemic necrosis of the fallopian tube and accompanying hydatid of Morgagni due to entrapment by adhesions. In the case in the present study, ischemic necrosis turned out to be reversible and the fallopian tube was preserved. However, due to the inflammation caused in the region, it was decided to only drain the hydatic of Morgagni instead of removing it in order to avoid further injury to the afflicted salpinx and preserve future fertility prospects. The presence of this cyst in this case, although notable, was not the primary etiology behind the observed pathology and was for the most part a concurrent condition.

The main limitation of the present study was its lack of involvement of multiple cases, which would allow for even a limited generalization of the findings. The present study paper describes a single case, believed to have not been previously described in the English literature. More valid conclusions could be drawn after conducting larger studies, aimed at investigating the etiology and risk factors associated with the development of postoperative pelvic adhesions, including the surgical techniques used.

In conclusion, surgical pelvic adhesions are a common complication following a cesarean section. The occurrence of preterm labor as a consequence of intra-abdominal inflammation caused by the entrapment of the fallopian tube with a hydatid of Morgagni in a postoperative pelvic adhesion is a unique case. However, in any instance of preterm labor onset in a pregnant woman with a history of previous cesarean section, the potential role of postoperative pelvic adhesions should be investigated as part of the mechanism of labor induction. The prenatal diagnosis of post-cesarean section adhesions is crucial for the prevention of severe intraoperative and post-operative complications, some of which are best treated in a tertiary medical center.

## Data Availability

The data used in the current study are available from the corresponding author upon reasonable request.
